# Spatial genetic patterns indicate mechanism and consequences of large carnivore cohabitation within development

**DOI:** 10.1002/ece3.4033

**Published:** 2018-04-17

**Authors:** Michael J. Evans, Tracy A. G. Rittenhouse, Jason E. Hawley, Paul W. Rego, Lori S. Eggert

**Affiliations:** ^1^ Wildlife and Fisheries Conservation Center Department of Natural Resources and the Environment University of Connecticut Storrs CT USA; ^2^ Wildlife Division Connecticut Department of Energy and Environmental Protection Sessions Woods WMA Burlington CT USA; ^3^ Division of Biological Sciences University of Missouri Columbia MO USA

**Keywords:** conservation genetics, landscape genetics, mammals, population ecology, wildlife management

## Abstract

Patterns of human development are shifting from concentrated housing toward sprawled housing intermixed with natural land cover, and wildlife species increasingly persist in close proximity to housing, roads, and other anthropogenic features. These associations can alter population dynamics and evolutionary trajectories. Large carnivores increasingly occupy urban peripheries, yet the ecological consequences for populations established entirely within urban and exurban landscapes are largely unknown. We applied a spatial and landscape genetics approach, using noninvasively collected genetic data, to identify differences in black bear spatial genetic patterns across a rural‐to‐urban gradient and quantify how development affects spatial genetic processes. We quantified differences in black bear dispersal, spatial genetic structure, and migration between differing levels of development within a population primarily occupying areas with >6 houses/km^2^ in western Connecticut. Increased development disrupted spatial genetic structure, and we found an association between increased housing densities and longer dispersal. We also found evidence that roads limited gene flow among bears in more rural areas, yet had no effect among bears in more developed ones. These results suggest dispersal behavior is condition‐dependent and indicate the potential for landscapes intermixing development and natural land cover to facilitate shifts toward increased dispersal. These changes can affect patterns of range expansion and the phenotypic and genetic composition of surrounding populations. We found evidence that subpopulations occupying more developed landscapes may be sustained by male‐biased immigration, creating potentially detrimental demographic shifts.

## 1 INTRODUCTION

As human development expands across Europe and North America, recovering populations of large carnivores increasingly interact with the human footprint (Chapron et al., 2014; Linnell, Swenson, & Anderson, 2001). Recently, shifting land‐use patterns from aggregated, high‐density (i.e., suburban) toward diffuse, low‐density (i.e., exurban) housing has changed the nature of these interactions (Brown, Johnson, Loveland, & Theobald, 2005). Although these land‐use patterns are at times associated with biodiversity loss and the introduction of exotic species (Bar‐Massada, Radeloff, & Stewart, 2014; Blair, 2004; Hansen et al., 2005), they can also provide habitat and resources to human‐adapted species (Johnston, 2001; McKinney, 2006). However, positive association of wildlife with human development (hereafter development) can mask changes to ecologically and evolutionarily important dynamics within populations occupying these landscapes (Remeš, 2000; Van Horne, 1983). For example, ecological traps can occur when species are attracted to habitat with negative demographic effects (Schlaepfer, Runge, & Sherman, 2002). Landscape modification also changes the abundance of resources and mortality sources, altering the selective landscape, which can change phenotypic distributions and evolutionary trajectories (Seehausen, Takimoto, Roy, & Jokela, 2008; Stronen et al., 2012). It is therefore important to the conservation and management of carnivores to understand how populations within development are maintained and identify shifts in population dynamics induced by development.

Historically, human land use has reduced and fragmented wildlife habitat (Fischer & Lindenmayer, 2007; Saunders, Hobbs, & Margules, 1991). Large carnivores have been particularly susceptible to these effects due to their low population densities, extensive ranges, and long generation times (Noss, Quigley, Hornocker, Merrill, & Paquet, 1996). Instead of removing habitat, exurban development often integrates low‐ and medium‐density housing within natural land cover (Clark, McChesney, Munroe, & Irwin, 2009; Stewart, Radeloff, Hammer, & Hawbaker, 2007). These patterns create intermixed landscapes facilitating greater exposure of animals to anthropogenic resources and mortality sources than binary urban‐wild systems (Bar‐Massada et al., 2014). While some carnivores may reach higher densities in developed areas (Evans, Hawley, Rego, & Rittenhouse, 2017; Fedriani, Fuller, & Sauvajot, 2001; Riley, Hadidian, & Manski, 1998), the ecological and evolutionary consequences of inhabiting development for large carnivore populations remain poorly known.

Features of development (e.g., roads and housing.) can alter dispersal and migration patterns that drive spatial genetic structure. Despite high mobility and dispersal potential, many large carnivores naturally exhibit significant spatial population structure, arising from both intrinsic and extrinsic factors (Rueness et al., 2003; Sacks, Brown, & Ernest, 2004). Many species display female natal philopatry (Waser & Jones, 1983) creating patterns of isolation by distance (IBD; Wright, 1943). Geographic, habitat, and anthropogenic barriers can also restrict the movement of dispersing individuals (McRae, Beier, Dewald, Huynh, & Keim, 2005; Millions & Swanson, 2007; Riley et al., 2006). Roads in particular are often avoided by carnivores and act as barriers to connectivity (Epps et al., 2005; Riley et al., 2006; Roever, Boyce, & Stenhouse, 2010). For large carnivores, roads may not directly limit movement, but can be an important source of additional mortality likely impacting more highly dispersive individuals, such as males and juveniles (Baker, Dowding, Molony, White, & Harris, 2007; Bateman & Fleming, 2012). Thus, a high prevalence of roads in intermixed landscapes can functionally limit dispersal and/or shift population demographics (Clark et al., 2009).

Genetic patterns reflecting migration and dispersal among carnivores inhabiting developed areas can identify the potential for ecological traps. Even if population densities are high, anthropogenic mortality can offset benefits, potentially forming sink populations solely maintained by immigration (Beckmann & Lackey, 2008). Alternatively, if resources provided by development outweigh anthropogenic mortality, subpopulations in these areas may become self‐sustaining and potentially act as sources of migrants supporting other surrounding populations (Hellgren, Onorato, & Skiles, 2005; Sweanor, Logan, & Hornocker, 2000; Weaver, Paquet, & Ruggiero, 1996). Thus, migration patterns between more rural, and more developed areas can be used to gauge whether subpopulations are sustained by immigration or recruitment (Andreasen, Stewart, Longland, Beckmann, & Forister, 2012; Ruiz‐Gonzalez, Cushman, Madeira, Randi, & Gómez‐Moliner, 2015). Asymmetrical gene flow can also provide mechanisms for human‐induced evolutionary changes. Elevated migration rates and distances can facilitate hybridization, outbreeding (Stronen et al., 2012), and gene swamping (Lenormand 2002). Identifying asymmetrical migration patterns can be critical in outlining metapopulation dynamics and predicting regional persistence, vulnerability, and future population expansion.

American black bears (*Ursus americanus*) are a prominent large carnivore occupying developed areas, and elevated bear densities in exurban relative to rural areas have recently been documented (Baruch‐Mordo et al., 2014; Evans et al., 2017; Johnson et al., 2015). However, inhabiting developed landscapes could alter the spatial genetic structure of bear populations in ways not yet understood. Black bears typically exhibit female philopatry—clusters of closely related females resulting from male‐biased dispersal (Rogers, 1987; Moore et al., 2014). These dispersal patterns are important in the avoidance of inbreeding (Costello, Creel, Kalinowski, Vu, & Quigley, 2008; Moyer, McCown, Eason, & Oli, 2006); thus, the degree to which features of development disrupt dispersal is important to the genetic health of populations (Beckmann & Lackey, 2008; Dixon et al., 2006; Hostetler et al., 2009). Spatial patterns of relatedness can also provide insight into the ecological processes underlying bear existence within development. Elevated densities may be maintained by an enrichment of anthropogenic resources facilitating increased overlap of unrelated individuals or reduced home range size (Atwood, Weeks, & Harmon, 2003; Horner & Powell, 1990; Mitchell & Powell, 2007; Vanak et al., 2013). With increasing overlap of unrelated individuals, patterns of IBD are expected to be less pronounced.

Our goal was to identify mechanisms explaining black bear persistence within developed areas and model changes in gene flow resulting from interaction with development. We previously identified higher bear densities and male‐biased sex ratios in exurban, relative to rural and suburban parts of this study area (Evans et al., 2017). Our first objective was to test the hypothesis that elevated bear densities in exurban areas would be associated with overlap of unrelated individuals, by quantifying differences in patterns of IBD. We predicted that female philopatry would be disrupted in more developed landscapes due to the prevalence of housing and roads. Our second objective was to test the hypothesis that populations in more developed areas are sustained by immigration by first identifying black bear spatial population structure and estimating the rate and directionality of migration between more rural and more developed areas. Finally, we used a landscape genetics approach (Manel, Schwartz, Luikart, & Taberlet, 2003)—testing for correlation between genetic similarity of individuals and characteristics of the intervening landscape—to identify how anthropogenic landscape features may facilitate or inhibit gene flow, contributing to observed and future patterns of bear distribution and genetic diversity. The absence of bear hunting in our study area allowed us to evaluate the role of landscape features on spatial genetic processes.

## 2 METHODS

### 2.1 Study area and sample collection

**Figure 1:**
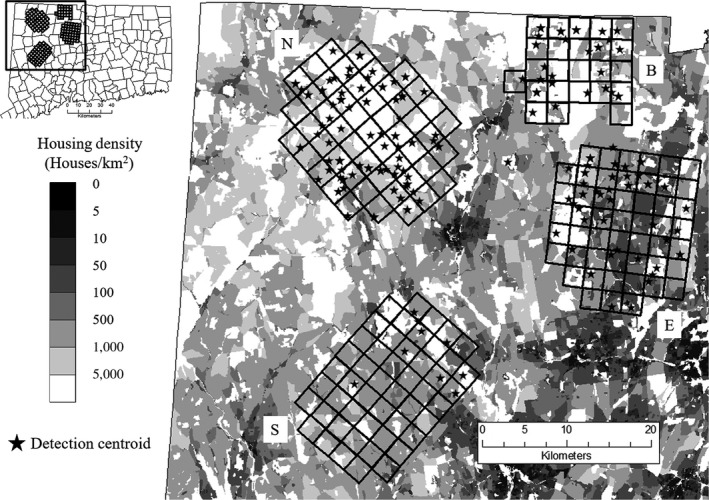
Locations of North (N), Barkhamsted (B), East (E), and South (S) sampling grids across black bear range in western Connecticut. Grids were used to distribute one noninvasive hair sampling station per cell across a gradient of housing densities in 2013 and 2014. The locations of individual black bears are represented by detection centroids (stars), calculated as the weighted mean of all locations at which an individual was detected

Noninvasive barbed wire hair corrals (Woods et al., 1999) were used to collect hair samples from black bears in northwest Connecticut (Evans et al., 2017). Corrals were constructed by stringing two strands of barbed wire around trees at 30 and 45 cm off of the ground forming an enclosure of ~5 × 5 m. We applied non‐nutritional scent lures to log piles at the center of corrals and to rags hung above corrals to attract bears. Hair corrals were distributed across sampling grids in four study areas (Figure 1) that encompassed most of black bear reproductive range in western Connecticut (hereafter CT), as determined by the CT Department of Energy and Environmental Protection (DEEP). Grid cells were 2.5 km^2^ to accommodate three to four sampling locations within an area the size of a typical female summer home range (approx. 30 km^2^, DEEP unpublished data). The northern grid (hereafter North grid) consisted of 49 sampling locations in the northwest corner of CT and covered 271 km^2^. Landcover in and around North grid was primarily forested, with mean housing density of 6.8 houses/km^2^. The eastern grid (hereafter East grid) contained 48 sampling locations across 215 km^2^ in and around suburban and exurban areas of CT, and mean housing density was 83.6 houses/km^2^. The southern grid (hereafter South grid) was 220 km^2^, contained 50 sampling sites, and was located in an attempt to span the southern extent of bear reproductive range (CT DEEP). Mean housing density within South grid was 23.2 houses/km^2^. The Barkhamsted grid was located at the northern boundary of CT and consisted of 25 sites over 95 km^2^. While similarly forested as North grid, mean housing density in Barkhamsted was 37.3 houses/km^2^.

Hair samples were collected every 7 days from June to August in 2013 and 2014 from most grids. Samples were only collected from Barkhamsted grid during 2014, while only the 25 northernmost corrals were sampled in South grid during 2014. Upon collection, all samples were stored in individually labeled coin envelopes collecting all hairs deposited on a single barb as a single sample. Scent lures were replenished and changed during each site visit. Within a sampling occasion, we applied the same lure at all sites.

### 2.2 Genetic methods

We extracted DNA from hair follicles using the InstaGene Matrix (Bio‐Rad Laboratories, Hercules, CA, USA) following the protocol of Eggert, Maldonado, and Fleischer (2005). DNA from a blood sample collected from a bear handled by CT DEEP during den visits was extracted using a DNeasy Blood and Tissue Kit (Qiagen, Valencia, CA, USA) and used as a positive control. Species identity was confirmed by amplifying a fragment of the mitochondrial cytochrome *b* region using the primers HCarn200 (Bidlack et al. 2007) and CanidL1 (Paxinos, McIntosh, Ralls, & Fleischer, 1997), followed by digestion with DDeII and APOI. Fragment sizes were visualized using gel electrophoresis, and we eliminated all samples not matching expected fragment lengths for bear.

Unique individuals were identified from hair samples using seven polymorphic microsatellite loci (G1A, G10B, G10L, G10P, G1D, G10M, G1C; Paetkau & Strobeck, 1994, 1998). We used the redesigned primer pairs of Kristensen, Faries, White, and Eggert (2011) to increase genotyping efficiency using low concentration and potentially degraded DNA from hair samples. All PCRs were performed in a UV‐sterilized hood following the multiplex genotyping protocol of Puckett et al. (2014). Amplified PCR products were cleaned of leftover enzymes by proteinase K digestion and then separated on an ABI 3730 DNA Analyzer (Applied Biosystems, Waltham, MA, USA) at the University of Missouri DNA Core Facility (Columbia, MO, USA). Individual genotypes were scored for each marker using GENEMARKER v1.97 (Soft Genetics, State College, PA, USA).

To confirm that markers had sufficient power to identify unique individuals, the *P*_(ID)sibs_ (Waits, Luikart, & Taberlet, 2001) was estimated in GENALEX (Peakall & Smouse, 2006). We used the multitubes approach (Taberlet et al., 1996) to produce consensus genotypes, amplifying and scoring three replicates of a sample to confirm homozygous genotypes, and heterozygous genotypes in at least two replicates. Only samples showing a consensus genotype in at least six loci were considered in further analyses. Individuals identified with the initial set of seven loci were then genotyped at an additional seven loci (G10J, G10O, P2H03, Mu05, Mu10, Mu23, and Mu59) to generate a 14‐loci genotype for all individuals used in further analyses. DROPOUT (McKelvey & Schwartz, 2005) was used to identify pairs of samples differing at three or fewer loci leading to misidentification of individuals. We regenotyped these samples and considered samples with a mismatch of adjacent alleles at one locus as recaptures of an individual. We then determined the sex of unique individuals by amplification of the amelogenin gene and visually scored products separated in agarose gel, as per Carmichael, Krizan, Blum, and Strobeck (2005).

We used GENEPOP (Raymond & Rousset, 1995) to test for deviation from Hardy–Weinberg equilibrium (HWE) and linkage disequilibrium (LD) among all loci within each study area. We used a sequential Bonferroni correction (Holm, 1979) to maintain a global α < .05, providing greater power to detect deviations while accounting for multiple comparisons (Rice, 1989). The presence of null alleles in each study area was assessed using MICROCHECKER (Van Oosterhout, Hutchinson, Wills, & Shipley, 2004). Both expected (*H*_E_) and observed (*H*_O_) heterozygosities, rarefaction‐adjusted allelic richness (*A*_R_), and inbreeding coefficients (*F*_IS_ *)* within each study area were estimated in FSTAT v2.9.3 (Goudet, 1995). FSTAT was also used to estimate the level of pairwise genetic differentiation (*F*_ST_) (Weir & Cockerham, 1984). We tested for differences in allelic richness between grids overall and per loci using the “diveRsity” (Keenan, McGinnity, Cross, Crozier, & Prodohl, 2013) package for R.

### 2.3 Spatial genetic structure

To identify the extent of spatial genetic structure and kin clustering within each study area, we estimated the extent of spatial autocorrelation of pairwise genetic relatedness (*r*) in GenAlEx v6.5 (Peakall & Smouse, 2006). This approach compares the pairwise geographic and squared individual genetic distance matrices to calculate an autocorrelation coefficient for each of a series of predetermined distance classes. Individual geographic locations were estimated using the centroids of minimum convex polygons formed by all hair corral locations visited by each individual (e.g., Coster & Kovach, 2012), hereafter detection centroids (Figure 1). We identified distance classes exhibiting significant, positive autocorrelation using 999 random permutations of genotypes among individuals, and 1,000 bootstrap estimates of *r*. Additionally, we compare the overall IBD trend using the strength of the relationship (*R* ^2^) between geographic and genetic distance on each grid.

Female dispersal distance was also compared between study areas as estimated by mean distance between individual centroids in parent–offspring relationships. We used ML‐RELATE (Kalinowski, Wagner, & Taper, 2006) to estimate the maximum likelihood probability of parent–offspring (PO), full‐sibling (FS), half‐sibling (HS), and unrelated relationships among all pairs of female individuals within each grid. Among pairs for which PO was the most likely relationship, we used a simulation of 999 permutations to test the probability of this relationship against the alternative hypothesis of FS. Pairs for which >90% of permutations indicated a higher likelihood of PO than FS were accepted as parent–offspring. We evaluated the accuracy of this procedure by applying it to 10 simulated dataset of 150 individuals with known relationships and report the error rate for all simulations. We then used a *t* test to compare mean dispersal distance between parent–offspring pairs on North and East grids.

We analyzed black bear population structure across northwest CT using the Bayesian assignment software STRUCTURE v2.3 (Pritchard, Stephens, & Donnelly, 2000). This program assigns individuals to one of *K* genetic groups by minimizing deviation from HWE at each locus and LD among loci within each group. We applied the admixture model with correlated allele frequencies option and performed 10 repetitions at values of *K* between 1 and 8 with a 10^6^ iteration burn‐in followed by 10^6^ sampling iterations. Replicates were averaged in CLUMPP v1.2 (Jakobsson & Rosenberg, 2007). We evaluated support for the number of genetic groups present using the log probability of the data, LnP(*K*) and the second‐order derivative rate of change in log probability, ∆*K* (Evanno, Regnaut, & Goudet, 2005), using STRUCTURE HARVESTER (Earl, 2012). We used STRUCTURE results to delineate putative genetic clusters occurring within CT for analysis of population scale movement patterns.

### 2.4 Recent migration rates

We quantified recent migration rates using BIMr 1.0 (Faubet & Gaggiotti, 2008) and GENECLASS (Piry et al., 2004). The F model implemented in BIMr allows for departure from HWE within populations, improving estimation of allele frequencies and producing accurate estimates of migration rates between weakly differentiated (*F*_ST_ > .01) populations (Faubet & Gaggiotti, 2008). Using study areas at which individuals were detected as putative population, we ran 10 replicates, each of which included 20 pilot runs of 1,000 iterations to optimize mixing parameters, followed by a 10^6^ iteration burn‐in. We then collected 10,000 samples from each replicate using a thinning interval of 1,000 iterations and examined parameter estimates from the run with the lowest Bayesian assignment deviance (*D*_assign_). We assessed migration asymmetry by examining 95% high‐density predictive intervals (HDPI) of posterior estimates of reciprocal migration rates for overlap and measuring the proportion of post‐burn‐in iterations at which a given migration rate estimate was greater than its reciprocal (Fordyce, Gompert, Forister, & Nice, 2011). To quantify potential differences in dispersal between sexes, we computed the Bayesian likelihood of first‐generation migrants for all individuals in GENECLASS 2. We simulated 10,000 genotypes and used a Type‐1 error rate cutoff of α = .05 to identify individuals assigned to genetic clusters other than their population of detection. Sex‐specific migration rates were estimated as the proportion of migrant individuals of each sex. We used a nonparametric chi‐square contingency test to assess the statistical significance of differences in proportions of individuals assigned to their study area of detection among areas, as well as reciprocal migration rates.

### 2.5 Landscape genetics

An individual‐based landscape genetics framework was used to compare patterns of IBD to those of isolation by landscape resistance (IBR) scenarios and identify features most likely influencing the spatial genetic structure of black bears in CT. Our modeling framework considered the potential effects of forest cover, roads, housing density, and combinations of these landscape features on black bear dispersal. We limited our analyses to females detected on North and East grid because females are the more philopatric sex and because the data for these grids include multiple years and represent the most disparate development context in our dataset. The general procedure followed a four‐step approach: creation of resistance surfaces, generation of pairwise resistance distance matrices, model fitting using resistance matrices as predictor variables, and comparing IBD to IBR scenarios.

#### 2.5.1 Resistance surfaces

First, we created landscape resistance surfaces in ArcMAP 10.1 (ESRI, Redlands, CA, USA) from reclassified land use and land cover data. To represent the effect of resistance due to forest cover, we created rasters from the Wildland Urban Interface (Radeloff et al., 2005) polygons, which provide percent forest cover per census block. Housing density was also rasterized using census block polygons from this same source (Radeloff et al., 2005). To represent the influence of roads, we rasterized line shapefiles from the topographically integrated geographic encoding and referencing (TIGER) database maintained by the United States Census Bureau (https://www.census.gov/geo/maps-data/data/tiger.html). Road‐based rasters assigned higher resistance values to cells corresponding to primary and secondary roads (TIGER Feature Classes S1100 and S1200), while remaining cells were given a value of one. Primary roads represent divided interstate and state highways accessible by interchanges, and secondary roads are major arteries in the United States, state, or county highway systems. Restricting our analysis to these types of roads eliminated local neighborhood, rural, and city streets, which are unlikely barriers as they occur frequently within bear home ranges in CT (Evans et al., 2017). All rasters representing hypothesized resistance surfaces were composed of 100 × 100 m cells, scaled from 0 to 100.

#### 2.5.2 Pairwise resistance distances matrices

We then used CIRCUITSCAPE v4.0 (http://www.circuitscape.org/; Shah & McRae, 2008) to create pairwise resistance distance matrices between all pairs of females across each resistance surface. Individual female locations were again represented by detection centroids. We chose to use landscape resistance, as opposed to least‐cost path analysis, because landscape resistance accounts for spatial heterogeneity in landscape composition, the possibility of multiple paths between two locations, and represents landscapes as continuous surfaces (McRae & Beier, 2007). It is more likely that bears experience landscapes as gradients of varying quality and movement resistance, rather than patch–matrix mosaics (Manning, Lindenmayer, & Nix, 2004 **;** McGarigal & Cushman, 2005). To evaluate support for each resistance surface, we used linear mixed models (LMM), applying the maximum likelihood population effects parameterization to account for the interdependence of pairwise data (Clarke, Rothery, & Raybould, 2002; Van Strien, Keller, & Holderegger, 2012). Relationships between scaled and centered resistance distances and individual genetic distances (estimated as *a*_r_; Rousset, 2000) were tested using the *lme4* package in R (R Core Team, 2014) and included a random effect for individual bears.

#### 2.5.3 Optimizing and modeling landscape features

We first used a univariate optimization procedure (Shirk, Wallin, Cushman, Rice, & Warheit, 2010; Spear, Balkenhol, Fortin, McRae, & Scribner, 2010) to identify the most supported relationship between each landscape variable and genetic distance. All resistance surfaces were rescaled to values ranging from 1 to 100. Continuous rasters of forest cover and housing density were rescaled using the equation from Shirk et al. (2010):

$$R={\left(V/V_{\max}\right)}^{x}\times 100.$$

Here, *R* is cell resistance, *V* is the original cell value, *V*_max_ is a constant representing the maximum allowed variable value, and *x* is a parameter determining response shape. For forest cover, *V*_max_ occurred at 1. For housing density, we set *V*_max_ at 500 houses/km^2^, a value consistent with our previous research defining the relationship between bear density and development in CT (Evans et al., 2017) and knowledge of black bear behavior around urban areas (Beckmann & Berger, 2003; Johnson et al., 2015; Merkle, Krausman, Decesare, & Jonkel, 2011). We also considered each variable as potentially facilitating dispersal to account for greater dispersal through unfavorable habitat (i.e., compensatory movement Knowlton & Graham, 2010; Peterman, Connette, Semlitsch, & Eggert, 2014). Therefore, we also generated inverse resistance surfaces using the equation:

$$R=(1-{\left(V/V_{\max}\right)}^{x})\times 100.$$

Primary and secondary roads were assigned values of 100, and the matrix was assigned a resistance value of 1 and vice versa to represent the inverse condition.

To account for potentially nonlinear responses, we represented different relationships between each variable and resistance by initially setting *x *=* *1 (i.e., linear response) then decreasing by 0.1 and increasing by one until a peak of support was identified (see: Shirk et al., 2010; Peterman et al., 2014). Model support was evaluated using AICc (Table 2). We then multiplied cell values in each of most supported univariate surface by 0, 1, 5, and 10 and generated multivariate resistance surfaces from all combinations of each weighted variable. Multivariate surfaces were then optimized combining the most supported representations of variably weighted roads, forest, and housing to assess the relative importance of each (Cushman, McKelvey, Hayden, & Schwartz, 2006; Spear et al., 2010). We identified the most supported model by fitting LMMs with fixed effects on pairwise resistance distance, using AICc.

#### 2.5.4 IBD versus IBR

Once we identified the most supported isolation by resistance model following univariate and multivariate optimization, we used a causal modeling framework (Cushman & Landguth, 2010; Cushman et al., 2006) to compare it to a model of IBD. To evaluate support for an IBR hypothesis, we compared *R* ^2^ and AICc weights from the corresponding LMM to a model with a fixed effect on the natural logarithm of Euclidean distance. We also fit a LMM including fixed effects on both resistance distance and Euclidean distance and used a modified $R_{B}^{2}$ statistic (Edwards, Muller, Wolfinger, Qaqish, & Schabenberger, 2008; Van Strien et al., 2012) to partial out the effects of Euclidean distance. For an IBR model to be supported, we expected greater AICc weight than the IBD model and a significant $R_{B}^{2}$ statistic controlling for Euclidean distance. Correlation coefficients were calculated using the Kenward‐Roger *F* and degrees of freedom (Kenward & Roger, 1997) estimated in the *pbkrtest* package (Halekoh & Højsgaard, 2014) in R.

## 3 RESULTS

### 3.1 Sampling results

We collected 814 hair samples in 2013 and 1226 hair samples in 2014, 935 of which were genetically determined to be black bear. Of these black bear samples, we successfully obtained individual genotypes for 734 samples. We found no more than two alleles per locus within a sample, supporting the assumption that hairs on any one barb came from only one individual. Our initial set of seven microsatellite loci provided sufficient power to distinguish unique individuals (*P*_ID_ = 5.2 × 10^−10^, *P*_IDsibs_ = 1.5 × 10^−4^). We identified 235 unique individuals and determined the sex of 198 bears (93 male, 105 female). We attribute the lower success rate of sex identification to potential sample degradation after as many as six previous genotyping reactions and the use of less sensitive scoring methods (i.e., visual bands) for amplified sex markers.

Among bears with at least three detections, mean distances between recaptures, as well as proportion of developed land cover encompassed by detection locations were highest on East grid and lowest on North grid (Table 1). On North grid, 48 of 49 sites produced bear hair, corresponding to 117 individuals (56 females and 47 males). On the East grid, detections at 37 of 48 sites corresponded to 62 individuals (29 females and 21 males). On Barkhamsted grid, 47 individuals (16 females and 20 males) were detected and bear hair was collected at 22 of 25 sites in 2014. Only 11 of the 50 South grid sites produced bear samples corresponding to 10 individuals (three females, six males, and one unknown). Due to the limited sample size, we report results only from STRUCTURE analyses for South grid. We did not detect the same individual on multiple sampling grids within a year. We detected one female and one male on East and Barkhamsted grids and one male on East and North grids between years.

**Table 1:** Measures of black bear genetic diversity and space use within study areas in western Connecticut. Metrics reported include number of individuals detected (*N*), allelic richness (*A*_R_), inbreeding coefficient (*F*_IS_), observed (*H*_O_) and expected (*H*_E_) heterozygosity, mean, standard deviation, and range of Rousset's genetic distance (*a*_r_) between individuals. Additionally, mean and standard deviation of distances between individual redetections (Dist) and proportion of developed land cover within areas encompassed by detection locations (%Dev)

	East	North	Barkhamsted
*N*	62	117	47
Dist (km)^a^	2.47 (3.23)	1.93 (3.36)	1.03 (1.59)
%Dev^a^	0.157	0.036	0.051
*F*_IS_	0.006 (*p* = .473)	0.029 (*p* = .041)	−0.009 (*p* = .613)
*A*_R_	5.54	6.85	5.48
*H*_O_	0.657	0.663	0.67
*H*_E_	0.661	0.683	0.654
*a*_r_	−0.027, 0.09 (−0.214 to 0.128)	−0.035, 0.005 (−0.079 to 0.202)	−0.014, 0.004 (−0.259 to 0.287)

a Measures calculated for individuals with at least three detections.

### 3.2 Genetic diversity

Two loci differed significantly from HWE in all study areas (G10L and P2H03) and were eliminated, creating 12‐loci genotypes for all analyses. Two loci differed significantly from HWE within the North grid only (Mu59; *p *=* *.001, and G10P; *p *=* *.003). As this pattern appeared in only one study area, we retained these loci for all analyses. Estimates of *F*_IS_ were 0.029 (*p *=* *.041) on North grid, 0.006 (*p *=* *.473) on East grid, and −0.009 (*p *=* *.391) on Barkhamsted grid, and genetic diversity was similar among study areas (Table 1). No loci used in analyses exhibited significant LD. *A*_R_ did not differ among grids overall (*p *>* *.10), although *A*_R_ of G10P and Mu59 was higher in North than East grid, *A*_R_ of Mu05 was higher in East than Barkhamsted, and *A*_R_ of G10J was higher in Barkhamsted than East. MICROCHECKER did not indicate evidence of deviation from HWE due to null alleles or genotyping error at any loci.

### 3.3 Spatial genetic structure

**Figure 2:**
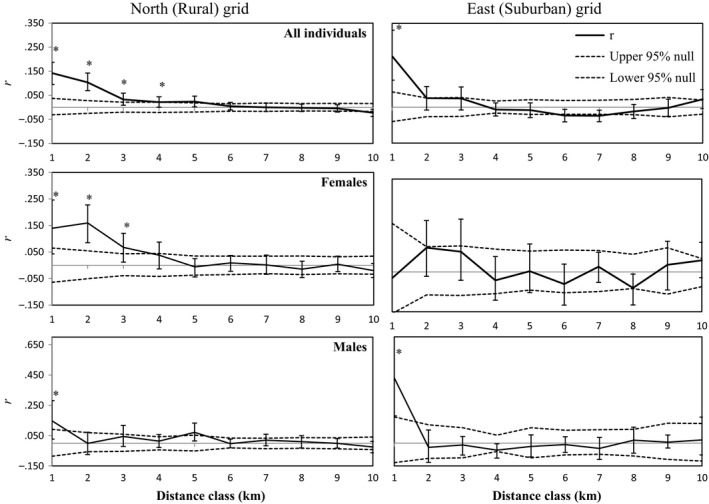
Correlogram plots depicting spatial autocorrelation of relatedness (*r*) among black bears detected on North and East grid in 2013 and 2014. Dashed lines indicate a 95% significance envelope surrounding the null hypothesis of randomly distributed genotypes, and vertical bars correspond to the 95% bootstrap distribution of r. Significant, positive autocorrelation (*) inferred by estimates falling outside of the null hypothesis envelope, and bootstrap distribution that does not overlap zero (solid gray line)

Spatial autocorrelation of genetic relatedness revealed differences in the extent of kin clustering between study areas and sexes. Black bears within North grid exhibited greater spatial genetic structure, compared to the East grid. Within North grid, there was a significant relationship between geographic distance and genetic distance (*R* ^2^ **= .15, *p *=* *.001) among females. We found significant, positive autocorrelation among North grid females with detection centers within 4 km (Figure 2). Females detected within the East grid did not exhibit a significant relationship between geographic distance and genetic distance (*R* ^2^ = .00196, *p *=* *.32), and none of the tested distance classes had significant positive autocorrelation (Figure 2). Males exhibited little spatial genetic structure in either study area. There was no relationship between geographic and genetic distance among males on either North (*R* ^2^ = .0098, *p *=* *.12) or East (*R* ^2^ = .01, *p *=* *.34) study areas. Males on North and East grids exhibited significant positive autocorrelation only within the closest (1 km) distance class considered (Figure 2). We identified 41 female parent–offspring pairs on North grid and 21 on East grid. The power of the ML‐RELATE approach to identify parent–offspring pairs was 0.72, with a 0.04 error rate using simulated data. Mean distance between individuals was higher (*p *=* *.07) on East grid (7,557 m, σ^2^ = 4,450 m) than North grid (6,556 m, σ^2^ = 4,393 m).

**Figure 3:**
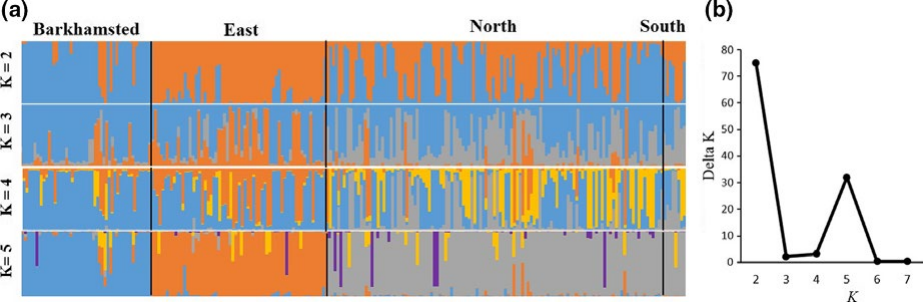
Population assignment probabilities to an a priori number of genetic clusters (*K*) produced by STRUCTURE (a) for all black bear individuals detected in northwest Connecticut 2013–2014. Vertical black bars separate individuals detected on Barkhamsted, East, North, and South study grids. Delta *K* values indicate the most likely number of clusters (b)

STRUCTURE results indicated support for *K *=* *2 and *K *=* *5 genetic clusters within the population of black bears in western Connecticut, based on peaks in *∆K* (Figure 3b). At *K *=* *2, individuals from East grid grouped as one genetic cluster, and individuals from Barkhamsted grid grouped as a second cluster. Individuals captured on North and South grids grouped with either the East or Barkhamsted clusters, with little admixture (Figure 3). At *K = *5, each grid was comprised primarily of individuals from clusters predominantly unique to those areas (South and North grid individuals grouped with the same cluster), with single individuals assigned to an outgroup (Figure 3). All three study areas were significantly differentiated from each other at the Bonferroni corrected α < .0167 and exhibited sufficient genetic differentiation to estimate recent migration rates using BIMr (*F*_ST_: 0.011–0.018).

### 3.4 Recent migration rates

**Figure 4:**
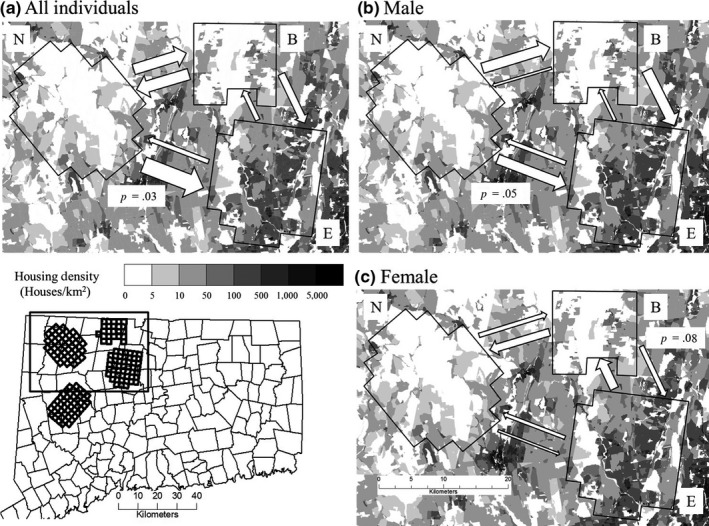
Black bear migration rates among all individuals, males, and females between study areas in northwest CT. Arrow sizes are proportional to first‐generation migration rates estimated by GENECLASS 2. Significant (*p* < .05) probabilities of asymmetry estimated by nonparametric chi‐square contingency tests are displayed for pairwise comparisons of migration rates between North (N), East (E), and Barkhamsted (B) grids

Estimates of migration rates were consistent among the 10 BIMr runs with the lowest Bayesian deviance (coefficient of variation: .005–.028). Migration rates estimated by the run with the lowest deviance had large HDPIs, which overlapped for all pairwise migration rates. Estimated migration rates between study areas for all individuals ranged from 3.5% of North individuals originating in East to 17.5% of Barkhamsted individuals originating in East (Table 2). Migration from North to East study areas was asymmetrical (*p *=* *.01). Proportions of first‐generation migrants estimated by GENECLASS 2 were also asymmetrical between North and East study areas (*p *=* *.03) and indicated sex‐specific asymmetries in migration. Proportions of individuals assigned to their study area of detection were significantly higher for North study area among all individuals and males (Table 2). Among females, migration was most frequent from East to Barkhamsted and from Barkhamsted to North study areas, whereas migration rates among males were highest from North to East and North to Barkhamsted study areas (Figure 4).

**Table 2:** Estimates of recent black bear migration rates between study areas in western Connecticut produced by BIMr and GENECLASS 2. BIMr estimates are the posterior means and 95% high‐density predictive interval from the run with the lowest Bayesian Deviance criterion (*D*_assign_). GENECLASS results are the proportion of individuals assigned to a study area. Bold values indicate reciprocal migration rates that were significantly (*p* < .05) different, indicating asymmetry. Asterisks denote study areas with significantly higher proportions of individuals assigned to their study area of detection

	From	To		
		East	North	Barkhamsted
BIMr				
All individuals	East	0.264 [0.095–0.615]	**0.035** [0.011–0.251]	0.073 [0.012–0.362]
	North	**0.132** [0.006–0.455]	0.335 [0.010–0.745]	0.136 [0.019–0.534]
	Barkhamsted	0.175 [0.018–0.652]	0.172 [0.016–0.528]	0.330 [0.026–0.866]
GENECLASS				
All	East	0.817	**0.027**	0.133
	North	**0.117**	0.910*	0.067
	Barkhamsted	0.067	0.064	0.800
Females	East	0.897	0.053	**0.125**
	North	0.034	0.840	0.063
	Barkhamsted	**0.069**	0.107	0.812
Males	East	0.810	**0.064**	0.100
	North	**0.143**	0.904*	0.150
	Barkhamsted	0.048	0.038	0.820

### 3.5 Landscape genetics

On East grid, the most supported univariate relationship between forest cover and genetic distance was an increasing exponential function (i.e. *x *=* *2) and a decreasing linear function (*x *=* *1) between housing density and genetic distance (Figure S1). A positive relationship between roads and genetic distance was more supported than a negative relationship (∆AICc = 3.72). Three multivariate IBR models combining these representations of forest cover, housing density, and roads met criteria as being more supported than an IBD model (lower AIC than an LMM of Euclidean distance and significant partial $R_{B}^{2}$ coefficients). All three of these models included resistance matrices representing the effect of decreasing resistance with increasing housing density and no other variables (Table 3).

**Table 3:** Results of linear mixed models relating multivariate landscape resistance to pairwise genetic distance among American black bears in western CT. Relative weights on landscape variables were 10 (high), 5 (medium), 1 (low), and 0. Model ∆AIC and partial $R_{B}^{2}$ coefficients controlling for the effect of Euclidean distance are reported. Analyses were performed among female individuals in North and East study areas. Results from resistance models with more support than a Euclidean distance (IBD) model and a significant $R_{B}^{2}$ from each study area are displayed

	Multivariate landscape model			$R_{B}^{2}$	*∆*AIC	$R_{B}^{2}$ *|*Distance
	House	Forest	Road			
East grid	Low	0	0	16.4^a^	0.000	34.2^b^
	Medium	0	0	16.4^a^	3.220	34.2^b^
	High	0	0	16.4^a^	3.272	34.2^b^
	Euclidean distance (IBD)			9.30	4.605	—
North grid	0	Low	Medium	37.9^b^	0.000	5.9^b^
	0	Low	High	37.9^b^	0.332	5.7^b^
	0	Low	Low	37.2^b^	0.870	5.5^b^
	0	0	Low	31.6^b^	1.519	0.6
	0	Low	0	35.9^a^	2.710	4.7^a^
	0	Medium	High	37.6^b^	3.540	5.8^b^
	0	Medium	Medium	37.2^b^	4.089	5.5^b^
	Euclidean distance			32.9^b^	9.839	–

a *p *<* *.05.

b *p *<* *.01.

On North grid, univariate resistance relationships were similar to East grid. An increasing linear (*x *=* *1) relationship between forest cover and genetic distance and a decreasing logarithmic relationship (*x *=* *0.3) between housing density and genetic distance were most supported (Figure S1). A positive relationship between roads and genetic distance was more supported (∆AICc = 0.00) than a negative relationship (∆AICc = 8.78). Seven multivariate IBR models met criteria as being more supported than an IBD model. None of these models included the effect of housing density. The four most supported (∆AICc > 2) included resistance matrices representing increasing resistance from roads and increasing forest cover, and the top two models indicated the relative resistance of roads was greater than forest cover (Table 3).

## 4 DISCUSSION

We found that intermixed development did not fragment a recolonizing population of black bears, but altered their spatial ecology in important ways. Spatial genetic patterns indicated anthropogenic features changed dispersal processes, potentially affecting population demographics and implicating landscape heterogeneity as a driver of dispersal. Female philopatry was disrupted around increased development, and greater distances between parent–offspring pairs in more developed areas indicate that greater interspersion of unrelated individuals may contribute to elevated bear densities (Evans et al., 2017). We identified a positive association between intervening housing density and genetic similarity between females, indicating increased gene flow through development in both rural and developed contexts. However, roads were associated with restricted gene flow only within the more rural study area, suggesting condition dependence in dispersal behavior that can affect population dynamics (Clobert, Galliard, Cote, Meylan, & Massot, 2009). Finally, asymmetrical male immigration into the more developed study area identified the potential for detrimental demographic shifts in these populations.

Weaker spatial autocorrelation of female relatedness (Figure 2) and greater female parent–offspring distances in East relative to North grid, despite similar genetic diversity and overall relatedness within the two areas (Table 1), indicated that female philopatry was disrupted around development. Previous work in this study area identified higher black bear densities in exurban relative to rural and suburban areas (Evans et al., 2017), and greater overlap of unrelated individuals could be one proximate mechanism maintaining this pattern. Although causal relationships cannot be drawn from two study areas, the result is consistent with anthropogenic foods increasing tolerance of unrelated conspecifics among black bears (Mitchell & Powell, 2007). While univariate LMMs indicated that high housing density facilitated gene flow among females in both North and East grids, only one census block within North grid had housing density >100 houses/km^2^, and an area of only 13.7 km^2^ had >50 houses/km^2^. Thus, housing density was unimportant, relative to forest cover and roads, in multivariate models of genetic distance among females on North Grid (Table 3). However, on the more developed East grid, where female philopatry was disrupted, housing density was the only important predictor of genetic distance (Table 3). These results suggest landscape heterogeneity caused by high‐density housing development may facilitate the breakdown of female philopatry.

Animals often move more quickly through nonhabitat than their preferred habitat, with generalists being more likely to disperse greater distances through nonpreferred habitat (Knowlton & Graham, 2010). The finding that female black bears living among development respond to high housing densities as inhospitable is consistent with recent research demonstrating selection for natural habitat when available among bears utilizing urban areas (Baruch‐Mordo et al., 2014; Johnson et al., 2015; Merkle, Robinson, Krausman, & Alaback, 2013). Greater distances between recaptures on East grid align with this conceptualization, and our landscape genetic results suggest a tendency for young female individuals to move quickly through development during dispersal. As spatial patterns of relatedness are determined by both dispersal behavior and the resulting fitness consequences, this response may diminish with age as bears become more adept at navigating intermixed landscapes (Johnson et al., 2015). Longer dispersal through high housing densities could affect human–wildlife dynamics, as it suggests range expansion may occur rapidly along urban–rural interfaces or through intermixed landscapes. Additionally, if anthropogenic foraging is socially learned (Breck, Lance, & Seher, 2009; Mazur & Seher, 2008) or heritable, the disproportionate emigration of individuals from developed contexts could accelerate propagation of these behaviors during range expansion.

Further, differences in the importance of roads in predicting genetic distance indicate context‐specific effects of landscape composition on female dispersal. In intermixed landscapes, roads can be the biggest contributor to fragmentation (Hawbaker, Radeloff, Clayton, Hammer, & Gonzalez‐Abraham, 2006). However, our results indicate that, while large roads were the most important features limiting gene flow in a rural context (among those considered), they did not present a dispersal barrier in a more developed context (Table 3). Roads may impede gene flow because bears avoid them, by demarcating home range boundaries (Coster & Kovach, 2012) or by elevating mortality. In rural contexts, black bears avoid high traffic volume roads (Carter, Brown, Etter, & Visser, 2010; Mitchell & Powell, 2007; Reynolds‐Hogland & Mitchell, 2007), often in response to increased vulnerability to hunting**.** As there is currently no bear hunting in CT, North grid individuals may avoid roads due to alternative risks, such as vehicle collisions (Bateman & Fleming, 2012; Beckmann & Lackey, 2008). The contrasting effects of roads on dispersal suggest that black bears in more developed areas either acclimate to roads to a greater degree than rural bears—possibly learning favorable crossing behavior from adults (Lewis et al., 2011; Mazur & Seher, 2008)—or experience high enough dispersal pressure that roads cannot be avoided.

These differences suggest that dispersal can be condition‐dependent and possibly related to variability in resource distribution. If dispersal has evolved to avoid inbreeding, one sex should remain strictly philopatric across conditions (Lawson & Perrin, 2007). The observed changes in philopatry between landscape contexts, in association with a negative relationship between housing density and relatedness, contradict this expectation. While male‐biased dispersal can effectively reduce inbreeding (Costello et al., 2008), our results build on recent work demonstrating context‐specific variability in black bear dispersal and mating systems (Moore, Xu, Frank, Draheim, & Scribner, 2015; Roy, Yannic, Côté, & Bernatchez, 2012), suggesting inbreeding avoidance may not be the evolutionary driver of dispersal behavior. Simulations have shown that, relative to other factors, inbreeding likely contributes very little selective pressure driving the evolution of dispersal (Guillaume & Perrin, 2006). Philopatry often evolves when habitat within the natal range is sufficiently productive, such that the potential fitness benefits of finding alternative habitat are outweighed by dispersal costs (Handley & Perrin 2007; Waser & Jones, 1983). Thus, the observed plasticity in dispersal behavior related to development supports the hypothesis that suitable habitat is more important than inbreeding avoidance and that female bears treat medium‐ to high‐intensity development as hostile or inhospitable habitat.

Longer female dispersal through development could produce asymmetrical female emigration from more to less developed areas, and estimated migration rates reflected this pattern (Figure 4). However, we did not sample adjacent populations to the north and west of our study areas, and some migrants in North and Barkhamsted grids from unsampled sources could have been erroneously identified as originating from East grid. At *K *=* *5, STRUCTURE results indicated eight individuals with high membership in a cluster that did not correspond to any study area (Figure 3a), likely representing an unsampled adjacent population. Overall, estimates indicated that East grid had the greatest proportion of individuals originating from other areas and asymmetrical immigration (Table 2). This result is less likely to be contaminated by unsampled sources, due to the location of East grid at the edge of bear range in CT. Additionally, we found evidence that immigration into the more developed grid was dominated by males (Figure 4). Both in the current study area and elsewhere, male‐skewed sex ratios have been observed around development (Beckmann & Lackey, 2008; Evans et al., 2017). Asymmetrical male immigration would explain these patterns, as sex‐specific dispersal characteristics can shift age structures and sex ratios toward young males (Cooley, Wielgus, Koehler, Robinson, & Maletzke, 2009; Robinson, Wielgus, Cooley, & Cooley, 2008). A prevalence of young males often has negative impacts on bear population growth rates (Costello, Creel, Kalinowski, Vu, & Quigley, 2009; Zedrosser, Bellemain, Taberlet, & Swenson, 2007). In conjunction with the implication that developed areas comprise marginal habitat, these detrimental demographics (i.e., elevated male sex ratios) illustrate the potential for areas of intermixed development to become ecological traps for black bears.

## 5 CONCLUSIONS

Our study illustrates the potential for intermixed development to covertly alter wildlife population dynamics and the importance of understanding these changes. Both in North America and globally, wildlife including large carnivores increasingly persists in proximity to development, outside of protected areas (Chapron et al., 2014; Linnell et al., 2001). Such circumstances may appear to indicate beneficial coexistence, yet intermixed development can induce changes with negative consequences for wildlife population demographics and human–wildlife dynamics. The volatility of land‐use patterns and anthropogenic mortality sources will also determine long‐term outcomes for such populations (Bettigole, Donovan, Manning, Austin, & Long, 2014; Clark et al., 2009). The altered dispersal behavior and sex ratios exhibited by a high‐density population of black bears living within development indicate shifted ecological dynamics that may constitute an ecological trap. We might expect similar responses to intermixed development among carnivores with large home ranges, as the effects of human disturbance on landscape connectivity are largely determined by species’ movement and perceptual abilities (Baguette & Van Dyck, 2007; Stevens, Verkenne, Vandewoestijne, Wesselingh, & Baguette, 2006).

## CONFLICT OF INTEREST

None declared.

## AUTHOR CONTRIBUTIONS

M.J.E., T.A.G.R, J.E.H., and P.W.R. designed the research. M.J.E., P.W.R., and J.E.H. collected the data. M.J.E. and L.S.E. conducted laboratory work. M.J.E. performed the analyses. M.J.E., L.S.E., and T.A.G.R interpreted the data and contributed to manuscript preparation.
