# Supplementary material for: Spatial genetic patterns indicate mechanism and consequences of large carnivore cohabitation within development
Source: Ecol Evol. 2018 Apr 17;8(10):4815–29. doi: 10.1002/ece3.4033 (PMC5980631; doi:10.1002/ece3.4033)
Supplement: Supplementary file 1 [file ECE3-8-4815-s001.docx]

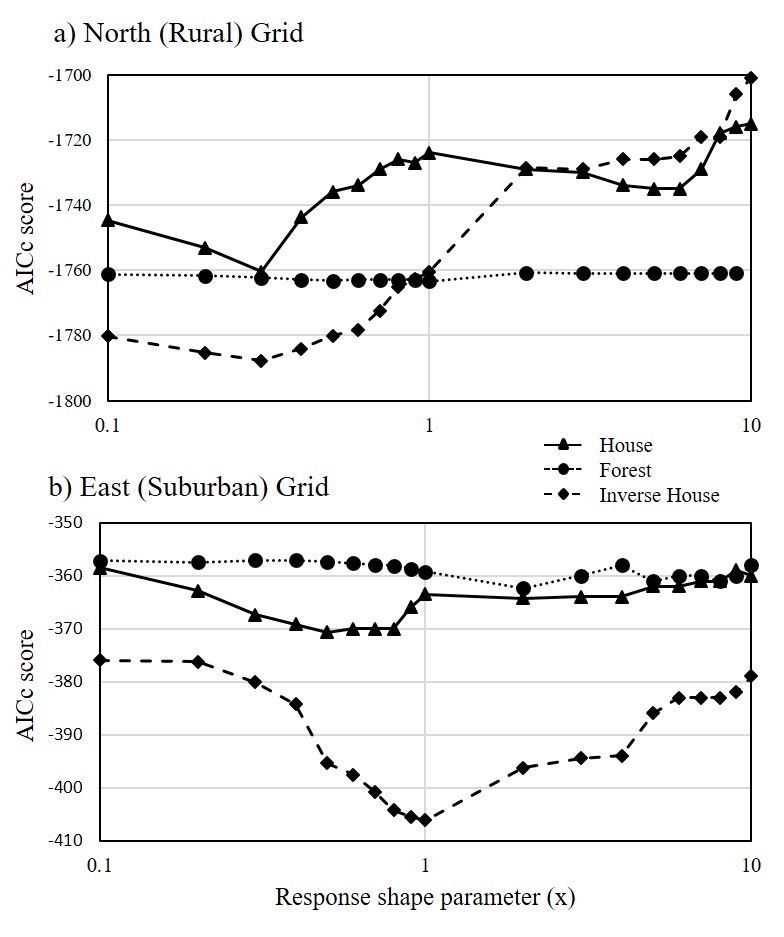


Fig. S1. Univariate resistance surface optimization results used to identify the most supported relationship between landscape variables and genetic distance on North and East study areas. Variables included housing density per census block (House), and percent forest cover within 1 km^2^ (Forest). Inverse relationships between landscape variables and genetic distance. Most-supported representations were identified by lowest AICc score. Inverse relationships between forest cover and genetic distance were ∆AICc > 200 in each area, and not shown.
